# Exploring hyperhidrosis and related thermoregulatory symptoms as a possible clinical identifier for the dysautonomic subtype of Parkinson’s disease

**DOI:** 10.1007/s00415-019-09325-w

**Published:** 2019-04-17

**Authors:** Daniel J. van Wamelen, Valentina Leta, Aleksandra M. Podlewska, Yi-Min Wan, Katarina Krbot, Elina Jaakkola, Pablo Martinez-Martin, Alexandra Rizos, Miriam Parry, Vinod Metta, Kallol Ray Chaudhuri

**Affiliations:** 1 King’s College London, Institute of Psychiatry, Psychology and Neuroscience, Department of Basic and Clinical Neurosciences, De Crespigny Park, London, SE5 8AF UK; 2 Parkinson Foundation Centre of Excellence, King’s College Hospital, Denmark Hill, London, SE5 9RS UK; 3 Radboud University Medical Centre, Department of Neurology, Donders Institute for Brain, Cognition and Behaviour, Nijmegen, The Netherlands; 4 Klinik fur Gerontopsychiatrie, Asklepios Nord-Ochsenzoll, Hamburg, Germany; 5 Division of Clinical Neurosciences, University of Turku, Turku, Finland; 6 National Centre of Epidemiology and CIBERNED, Carlos III Institute of Health, Madrid, Spain

**Keywords:** Parkinson’s disease, Hyperhidrosis, Autonomic, Non-motor symptoms, Dyskinesia

## Abstract

**Objective:**

To identify associated (non-)motor profiles of Parkinson’s disease (PD) patients with hyperhidrosis as a dominant problem.

**Methods:**

This is a cross-sectional, exploratory, analysis of participants enrolled in the Non-motor Longitudinal International Study (NILS; UKCRN No: 10084) at the Parkinson’s Centre at King’s College Hospital (London, UK). Hyperhidrosis scores (yes/no) on question 28 of the Non-Motor Symptom Questionnaire were used to classify patients with normal sweat function (*n* = 172) and excessive sweating (*n* = 56) (Analysis 1; *n* = 228). NMS scale (NMSS) question 30 scores were used to stratify participants based on hyperhidrosis severity (Analysis 2; *n* = 352) using an arbitrary severity grading: absent score 0 (*n* = 267), mild 1–4 (*n* = 49), moderate 5–8 (*n* = 17), and severe 9–12 (*n* = 19). NMS burden, as well as PD sleep scale (PDSS) scores were then analysed along with other correlates.

**Results:**

No differences were observed in baseline demographics between groups in either analysis. Patients with hyperhidrosis exhibited significantly higher total NMSS burden compared to those without (*p* < 0.001). Secondary analyses revealed higher dyskinesia scores, worse quality of life and PDSS scores, and higher anxiety and depression levels in hyperhidrosis patients (*p* < 0.001). Tertiary analyses revealed higher NMSS item scores for fatigue, sleep initiation, restless legs, urinary urgency, and unexplained pain (*p* < 0.001).

**Conclusions:**

Chronic hyperhidrosis appears to be associated with a dysautonomia dominant subtype in PD patients, which is also associated with sleep disorders and a higher rate of dyskinesia (fluctuation-related hyperhidrosis). These data should prompt the concept of hyperhidrosis being used as a simple clinical screening tool to identify PD patients with autonomic symptoms.

## Introduction

Hyperhidrosis is one of the least studied non-motor symptoms (NMS) in Parkinson’s disease (PD). Sweating disorders in PD include mainly hyperhidrosis, but also hypohydrosis, and the prevalence is reported by some at 64% of PD patients, compared to 12.5% of healthy controls [1]. Phenomenologically, hyperhidrosis in PD could be chronic or paroxysmal, on the one hand, and non-fluctuating or related to non-motor fluctuations, on the other [2]. The phenomenon of hyperhidrosis may also overlap with an increase of sebum excretion rate known as seborrhoea which is also frequent in PD patients [3]. The pathophysiology remains unclear but dysautonomia has been suggested as a possible correlate, while sweating occurs both during levodopa-induced motor fluctuations [4] and dyskinesia [5]. The pattern of dyshidrosis in PD appears to differ from the general population and axial hyperhidrosis in PD is associated with decreased activation of sweat glands in the palms of the hands suggesting that axial hyperhidrosis could be a compensatory phenomenon for reduced sympathetic function in the extremities [5].

Recently, Sauerbier et al. and Marras and Chaudhuri [6, 7] proposed several non-motor subtypes in PD, analogues to the better-known motor subtypes. One of these phenotypes is an autonomic phenotype, with adrenergic/cholinergic features (such as orthostatic hypotension) and gastro-intestinal symptoms overlapping with sleep dysfunction and levodopa-induced dyskinesias [6–8]. Here, we investigate, using validated tools (Non-Motor Questionnaire and Scale), whether in PD patients with self-declared hyperhidrosis specific patterns of the autonomic subtype of PD exist.

## Methods

This is a post hoc, exploratory study using the Non-motor International Longitudinal Study’s (NILS) international database. We used data from patients clinically diagnosed with idiopathic PD and whose data were entered between November 2011 and September 2018. We only included the baseline assessments (of five yearly longitudinal assessments) of patients seen at King’s College Hospital London (NHS Foundation Trust). The NILS Study is the world’s first non-motor focussed comprehensive longitudinal cohort study addressing non-motor profiling of PD and the natural history of non-motor symptoms together with treatment response and clinico-pathological correlations. The study is adopted as a national study by the National Institute of Health Research in the UK (UKCRN No: 10084) and involves 14 centres across Europe, but for the current study only patients who had a baseline assessment at King’s College Hospital were included. The study was authorised by local ethics committees (NRES SouthEast London REC3, 10084, 10/H0808/141). All patients gave written consent prior to study procedures in accordance with the Declaration of Helsinki. Exclusion criteria were (1) diagnosis of Parkinsonism different to idiopathic PD; (2) dementia (as per internationally accepted criteria); and (3) inability for giving consent to participate in the study.

Data extracted from the NILS database concerned sex, disease onset and duration (in years), and Levodopa equivalent dose (LEDD). Patient-reported outcomes were: Hospital Anxiety and Depression Scale (HADS; a 14-item, patient-completed scale with subscales for anxiety and depression) [10]; PDQ-8 (a specific instrument for assessment of health-related quality of life in PD) [11]; PD Sleep Scale-version 1 (PDSS), a 15-item, patient-completed clinical tool used to assess the frequency of sleep disturbances during the past week in PD patients) [12], and Epworth Sleepiness Scale (ESS) scores (6-item tool looking at daytime sleepiness) [13]. Clinician-based evaluations were: Hoehn and Yahr (HY) staging [9] and Non-Motor Symptoms Scale (NMSS). The NMSS considers the frequency and severity of the non-motor symptoms of PD grouped into nine domains: cardiovascular, sleep/fatigue, mood/apathy, perceptual problems/hallucinations, attention/memory, gastrointestinal tract, urinary function, sexual function, and miscellaneous [14, 15].

Hyperhidrosis scores on question 28 of the validated Non-Motor Symptom Questionnaire (NMSQ; when available) were used to classify patients with normal sweat function (*n* = 172) and hyperhidrosis (*n* = 56) (analysis 1). In addition, we used the NMSS question 30 scores to stratify participants based on hyperhidrosis severity (analysis 2): absent score 0 (*n* = 267), mild scores 1–4 (*n* = 49), moderate scores 5–8 (*n* = 17), and severe scores 9–12 (*n* = 19). Subsequently, we assessed differences in NMS burden measured on the NMSS. Secondary and tertiary outcomes included differences in specific non-motor (HADS, PDSS, ESS) and motor scores. As this was an exploratory analysis, we did not perform cluster analysis. Group differences were tested with Mann–Whitney *U* test or Kruskal–Wallis test, where appropriate. A Bonferroni correction was used to correct for multiple testing for the secondary (*p* = 0.05/8) and tertiary (*p* = 0.05/30) outcomes. To test for gender differences, Pearson Chi-square analysis was used. For correlations, Spearman’s analysis was used. All data were analysed using SPSS Version 24 (IBM SPSS Statistics for Windows, Version 24.0. Armonk, NY: IBM Corp.).

## Results

No differences were observed in age, gender, disease duration, and Levodopa Equivalent Dose (LED) between patients with and without hyperhidrosis (analysis 1). There were also no baseline differences in the NMSS defined groups (analysis 2), except a trend for LED (*p* = 0.017; Table 1). 160 of the 172 (93%) patients who indicated no sweating on the NMSQ had no hyperhidrosis on the NMSS scale (absent score) confirming the high concordance between the NMSQ and NMSS.

**Table 1:** Demographics and (non-)motor profiles (non-motor symptom questionnaire based) associated with hyperhidrosis in Parkinson’s disease

Analysis 1 (NMSQ item 28)			
Hyperhidrosis	No (*n* = 172)	Yes (*n* = 56)	*P*
Demographics			
Age (years)	64.7 ± 11.4	62.6 ± 10.8	0.20
Gender (M/F)	119/53	36/20	0.50
Disease duration (years)	5.0 ± 4.9	5.7 ± 4.7	0.30
LED (mg)	522.9 ± 440.0	663.5 ± 545.5	0.09
NMSS total	41.6 ± 33.0	73.4 ± 48.8	**< 0.001**
1. Dizziness	1.2 ± 2.2	1.9 ± 2.8	0.08
2. Falls	0.3 ± 1.2	0.8 ± 2.1	0.019
3. Somnolence	2.0 ± 3.0	2.8 ± 3.9	0.15
4. Fatigue	2.6 ± 3.1	4.4 ± 4.4	0.009
5. Sleep initiation	2.7 ± 3.7	4.3 ± 4.5	0.020
6. Restless legs	1.5 ± 2.7	3.0 ± 3.8	**0.001**
7. Loss of interest	1.1 ± 2.5	2.0 ± 3.5	0.10
8. Motivation	1.2 ± 2.5	2.3 ± 3.7	0.19
9. Feeling nervous	1.1 ± 2.7	2.9 ± 3.8	0.005
10. Depression	1.7 ± 2.8	3.2 ± 3.8	0.002
11. Flat moods	1.3 ± 2.3	1.6 ± 2.8	0.64
12. Anhedonism	1.1 ± 2.4	1.6 ± 3.3	0.64
13. Hallucinations	0.5 ± 1.4	0.8 ± 2.2	0.42
14. Delusions	0.2 ± 1.0	0.4 ± 1.6	0.80
15. Diplopia	0.3 ± 1.1	0.9 ± 2.6	0.25
16. Concentration	1.3 ± 2.3	2.1 ± 2.9	0.049
17. Recall	1.5 ± 2.5	2.6 ± 3.0	0.006
18. Forgetfulness	1.3 ± 2.3	1.8 ± 2.5	0.17
19. Sialorrhoea	1.2 ± 2.3	2.2 ± 3.3	0.018
20. Dysphagia	0.7 ± 1.8	1.4 ± 2.5	0.013
21. Constipation	1.6 ± 2.5	3.1 ± 4.2	0.047
22. Urgency	2.4 ± 3.5	4.7 ± 4.6	**0.001**
23. Frequency	1.6 ± 3.0	2.9 ± 3.9	0.036
24. Nocturia	2.7 ± 3.6	3.2 ± 4.2	0.72
25. Libido	1.4 ± 2.9	1.9 ± 3.3	0.10
26. Performing sex	1.3 ± 3.0	1.3 ± 2.9	0.68
27. Pain	1.4 ± 2.7	3.7 ± 4.2	**< 0.001**
28. Anosmia	2.7 ± 3.7	4.1 ± 4.7	0.046
29. Weight change	0.7 ± 2.0	1.4 ± 3.2	0.28
30. Hyperhidrosis	–	–	–
PDSS	109.7 ± 27.2	98.1 ± 28.2	**0.006**
ESS	7.4 ± 4.7	9.0 ± 6.0	0.16
HADS anxiety	5.3 ± 3.8	8.1 ± 4.3	**< 0.001**
HADS depression	4.9 ± 3.9	7.0 ± 4.1	**< 0.001**
PDQ-8	7.1 ± 5.4	11.7 ± 7.4	**< 0.001**
SCOPA-motor	10.3 ± 5.0	11.4 ± 5.2	0.13
SCOPA-daily living	5.1 ± 3.3	6.8 ± 4.4	0.019
SCOPA-complications	1.5 ± 1.9	3.0 ± 3.3	**0.002**

Statistical differences tested using Mann–Whitney *U* test

*M* male, *F* female, *LED* Levodopa equivalent dose, *PDSS* Parkinson’s disease sleep scale, *ESS* Epworth sleepiness scale, *HADS* hospital anxiety and depression scale, *PDQ-8* 8-item Parkinson’s disease quality of life scale, *SCOPA* Scales for outcomes in Parkinson’s disease

**Fig. 1:**
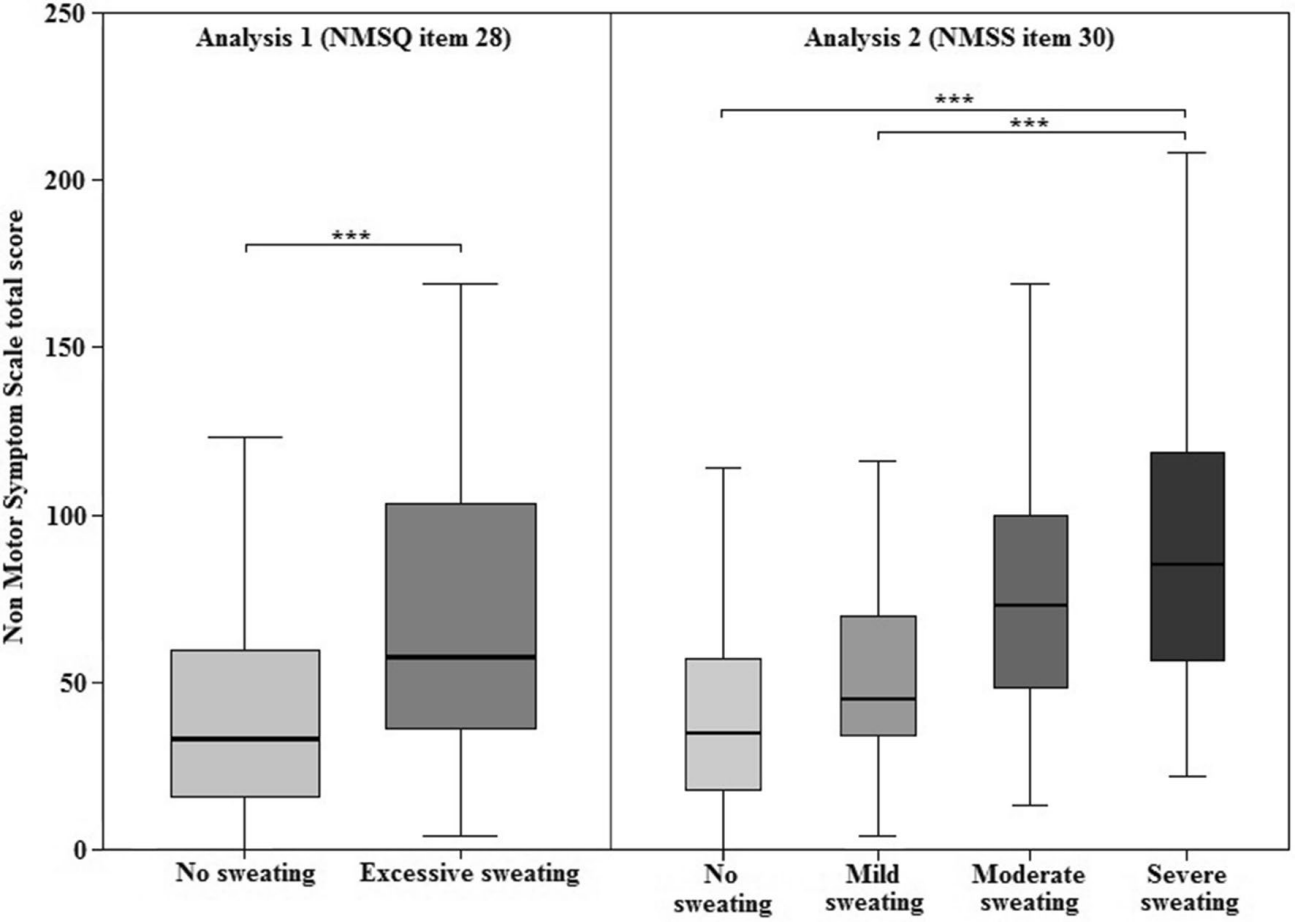
Non-motor symptom Scale total scores differences between Parkinson’s disease patients with hyperhidrosis and no hyperhidrosis and across the different severities of hyperhidrosis. *NMSQ* Non-motor symptom questionnaire, *NMSS* Non-motor symptom scale, ***p < 0.001. Statistical differences tested using Mann–Whitney *U* and Kruskal–Wallis tests

In both analyses, patients with hyperhidrosis exhibited significantly higher NMSS total scores (analysis 1: 73.4 vs 41.6; *p* < 0.001 and analysis 2: 41.9 for absent, 58.2 for mild, 77.8 for moderate, and 93.7 for severe; *p* < 0.001) (Fig. 1; Tables 1 and 2). Secondary analyses revealed, after correction for multiple testing, significantly higher Scales for Outcomes in PD (SCOPA) dyskinesia scores in hyperhidrosis patients (*p* < 0.001 for analysis 1 and *p* = 0.001 for analysis 2), worse quality of life scores (PDQ-8; *p* < 0.001 both analyses), worse PDSS (*p* = 0.006 for analysis 1 and *p* < 0.001 analysis 2) and higher anxiety levels (*p* < 0.001 for analysis 1 and *p* = 0.003 for analysis 2) and depression levels (*p* < 0.001 for analysis 1) on the Hospital Anxiety and Depression Scale (Tables 1 and 2). In addition, we correlated hyperhidrosis severity (defined by NMSS item 30) with SCOPA dyskinesia scores, and also with SCOPA fluctuation presence and severity subitem scores. The analysis showed a significant positive correlation between hyperhidrosis severity and dyskinesia (for both SCOPA dyskinesia total scores, and subitems 20 and 21 for fluctuation presence and severity; rho ≥ 0.162; *p* ≤ 0.002).

**Table 2:** Demographics and (non-)motor profiles (non-motor symptom scale based) associated with hyperhidrosis in Parkinson’s disease

Analysis 2 (NMSS item 30)					
Hyperhidrosis	No (*n* = 267)	Mild (*n* = 49)	Moderate (*n* = 17)	Severe (*n* = 19)	*P*
Demographics					
Age (years)	64.5 ± 11.6	63.4 ± 11.4	60.8 ± 11.4	62.3 ± 11.5	0.61
Gender (M/F)	183/84	33/16	12/5	8/11	0.13
Disease duration (years)	5.2 ± 5.1	6.3 ± 5.6	4.2 ± 3.2	6.9 ± 3.8	0.033
LED (mg)	522.7 ± 467.2	613.4 ± 477.6	631.5 ± 473.8	815.3 ± 460.0	**0.017**
NMSS total	41.9 ± 32.3	58.2 ± 39.6	77.8 ± 41.6	93.7 ± 52.6	**< 0.001**
1. Dizziness	1.4 ± 2.2	1.5 ± 2.5	1.9 ± 3.2	2.7 ± 3.3	0.31
2. Falls	0.4 ± 1.3	0.4 ± 1.2	0.4 ± 1.2	1.4 ± 3.0	0.028
3. Somnolence	1.7 ± 3.0	2.4 ± 3.1	2.1 ± 3.3	3.9 ± 4.2	0.014
4. Fatigue	2.4 ± 3.1	4.2 ± 3.9	5.0 ± 3.7	6.1 ± 4.6	**< 0.001**
5. Sleep initiation	2.7 ± 3.6	3.5 ± 3.6	4.9 ± 4.2	6.9 ± 4.1	**< 0.001**
6. Restless legs	1.6 ± 2.8	1.8 ± 2.0	4.2 ± 3.9	3.7 ± 3.8	**< 0.001**
7. Loss of interest	1.1 ± 2.4	1.3 ± 2.6	3.4 ± 4.1	1.1 ± 3.0	0.31
8. Motivation	1.1 ± 2.4	1.8 ± 2.8	2.2 ± 4.1	2.1 ± 3.5	0.15
9. Feeling nervous	1.7 ± 2.9	2.3 ± 3.1	2.5 ± 3.6	3.7 ± 4.5	0.31
10. Depression	1.7 ± 2.8	2.7 ± 3.3	3.5 ± 3.3	2.5 ± 3.9	0.005
11. Flat moods	1.2 ± 2.2	1.7 ± 2.7	1.9 ± 2.7	1.7 ± 3.2	0.37
12. Anhedonism	0.9 ± 2.2	1.9 ± 3.3	1.4 ± 3.0	1.0 ± 2.8	0.008
13. Hallucinations	0.5 ± 1.5	0.5 ± 1.3	0.6 ± 1.37	1.6 ± 3.9	0.78
14. Delusions	0.2 ± 0.8	0.5 ± 1.7	0.6 ± 1.7	0.9 ± 3.0	0.86
15. Diplopia	0.4 ± 1.4	0.5 ± 1.9	1.3 ± 3.4	0.3 ± 1.4	0.66
16. Concentration	1.5 ± 2.4	1.7 ± 2.6	1.8 ± 2.1	2.8 ± 4.1	0.83
17. Recall	1.6 ± 2.4	2.1 ± 2.7	2.1 ± 2.0	3.2 ± 3.8	0.09
18. Forgetfulness	1.4 ± 2.4	1.7 ± 2.6	1.7 ± 2.4	2.1 ± 2.9	0.53
19. Sialorrhoea	1.1 ± 2.4	1.6 ± 2.8	0.8 ± 2.0	3.2 ± 4.0	0.049
20. Dysphagia	0.7 ± 1.8	1.4 ± 2.8	1.5 ± 2.2	0.6 ± 1.1	0.11
21. Constipation	1.9 ± 2.9	2.1 ± 3.2	1.9 ± 3.2	3.9 ± 4.5	0.05
22. Urgency	2.4 ± 3.5	3.4 ± 4.2	4.3 ± 4.9	4.8 ± 4.6	0.029
23. Frequency	1.7 ± 2.9	2.2 ± 3.6	1.9 ± 4.0	4.6 ± 4.2	0.003
24. Nocturia	2.6 ± 3.4	2.6 ± 3.6	3.5 ± 4.2	4.1 ± 7.7	0.28
25. Libido	1.3 ± 2.7	1.3 ± 2.8	3.4 ± 4.4	2.0 ± 3.3	0.68
26. Performing sex	1.2 ± 2.8	1.3 ± 2.9	2.3 ± 4.4	1.7 ± 3.6	0.21
27. Pain	2.0 ± 3.2	2.8 ± 3.6	4.7 ± 4.7	3.5 ± 4.6	0.004
28. Anosmia	2.8 ± 3.8	3.4 ± 4.1	5.4 ± 4.4	5.33 ± 4.7	0.004
29. Weight change	0.7 ± 2.0	1.0 ± 2.0	1.4 ± 4.0	2.3 ± 4.2	0.08
30. Hyperhidrosis	–	–	–	–	–
PDSS	109.2 ± 26.6	95.1 ± 31.1	95.9 ± 22.3	89.4 ± 23.0	**< 0.001**
ESS	7.4 ± 4.8	8.5 ± 5.9	8.9 ± 6.0	9.6 ± 5.8	0.44
HADS anxiety	5.8 ± 3.9	7.6 ± 3.8	7.6 ± 4.3	8.4 ± 5.1	**0.003**
HADS depression	5.3 ± 3.7	6.3 ± 3.7	7.5 ± 5.0	6.5 ± 3.3	0.043
PDQ-8	7.7 ± 5.9	11.1 ± 6.6	10.5 ± 6.3	9.6 ± 5.8	**< 0.001**
SCOPA-motor	10.4 ± 5.4	11.5 ± 6.5	12.1 ± 4.4	13.9 ± 6.5	0.047
SCOPA-daily living	5.5 ± 3.7	6.1 ± 4.0	6.5 ± 4.1	8.1 ± 4.8	0.08
SCOPA-complications	1.7 ± 2.2	2.8 ± 3.1	2.1 ± 1.7	4.4 ± 3.6	**0.001**

Statistical differences tested using Kruskal–Wallis test

*M* male, *F* female, *LED* Levodopa equivalent dose, *NMSS* non-motor symptom scale, *PDSS* Parkinson’s disease sleep scale, *ESS* Epworth sleepiness scale, *HADS* hospital anxiety and depression scale, *PDQ-8* 8-item Parkinson’s disease quality of life scale, *SCOPA* Scales for Outcomes in Parkinson’s disease

Tertiary analyses, corrected for multiple testing, revealed higher NMSS scores for restless legs, urinary urgency and pain (analysis 1; *p* < 0.001; Table 1) and for fatigue, sleep initiation, and restless legs (analysis 2; *p* < 0.001), with nearly significant trends for urinary frequency, pain and anosmia (analysis 2; *p* = 0.004; Table 2). Moreover, there were significant positive correlations between hyperhidrosis severity and fatigue, sleep initiation, restless legs, hyposmia, and weight change (rho ≥ 0.177; *p* < 0.001), with statistical trends for daytime sleepiness, depression, and urinary urgency (rho ≥ 0.158; *p* = 0.003).

## Discussion

In this exploratory study, we observed a significantly higher NMS burden in PD patients with hyperhidrosis, especially among those with the most severe hyperhidrosis. Moreover, PD patients exhibiting hyperhidrosis have higher dyskinesia scores, worse quality of life, more severe sleep disorders, and higher anxiety and depression levels. The autonomic phenotype of this subset was characterised by NMS such as sleep, depression, anxiety, urinary problems and pain. Based on these results, the presence and severity of hyperhidrosis could be used as simple clinical screening tool to support the presence of this specific subtype in PD.

The above statement is underpinned by the fact that hyperhidrosis in our cohort closely resembles the autonomic phenotype that has been previously proposed in PD [6]. Clinically, it has been proposed that this PD subtype presents with dysautonomia, such as sexual dysfunction, constipation, urinary frequency and urgency. Other symptoms included in this phenotype are postural and post-prandial hypotension, and sialorrhoea [16]. Also fatigue, which was significantly more severe in our cohort among patients suffering from hyperhidrosis, has been implicated in this subtype of PD [6].

Identifying distinct subtypes in PD is of great importance for therapeutic and prognostic reasons and forms the basis of the emerging concept of holistic personalised medicine strategies for PD. It has been shown that PD patients with autonomic dysfunction in general, but also individual autonomic abnormalities, such as sweating disorders, show a more rapid disease progression and shorter survival compared to those without such abnormalities [17]. Moreover, hyperhidrosis is associated with pain and decreased quality of life [16]. Whether treatment of autonomic symptoms and hyperhidrosis leads to slowing down of the disease progression and improvement of survival remains currently unclear. Moreover, due to the lack of validated and evidence-based algorithms, the treatment of hyperhidrosis in PD patients remains challenging, and its effectiveness limited.

The only motor difference between PD patients suffering from hyperhidrosis and those without was an increased severity of dyskinesia. It might be argued that, from a mechanistic point of view, dyskinesia itself leads to increased sweating. However, a recent meta-analysis on the risk of excessive sweating with antidepressants showed that hyperhidrosis is associated with increased dopamine receptor affinity of specific antidepressants [18], underlining the link between hyperhidrosis and dopamine metabolism. Other explanations could be found in the hypothalamus, one of the key structures in thermoregulation. It is known that the hypothalamus is affected by Lewy pathology in PD [19] and also the hypothalamic functional connectivity is disturbed in autonomic dysfunction in PD [20]. Furthermore, hyperhidrosis in PD is often episodic [18] and similar sweat attacks occur during menopause, where they have been related to brain noradrenergic over activity [21]. Perhaps, similar changes to the noradrenergic system occur in PD, explaining hyperhidrosis.

Limitations in this study include the cross-sectional design and retrospective data mining, rather than a longitudinal follow-up to assess the development of specific symptoms in PD patients suffering from hyperhidrosis. In addition, we did not assess symptoms related to impaired thermoregulation, and dehydration, often related to prominent dyskinesias [22]. Also, the presence and severity of hyperhidrosis were only assessed through history taking and not objectively assessed. Moreover, both NMSS and NMSQ contain only one item each addressing hyperhidrosis [14, 15]. The NMSS and NMSQ are, however, validated tools for assessing NMS in PD patients and are commonly used tools in outpatient clinics, reflecting a real-world experience. As such we feel that our current results are useful, also considering that there were no group differences regarding disease duration, Hoehn and Yahr stages, LED and sex which in themselves can also influence NMS burden and dyskinesia severity.

In summary, the presence and severity of hyperhidrosis in PD patients coincides with an apparent PD endophenotype with dysautonomia which is associated with sleep disorders and a higher rate of dyskinesia. We suggest that asking about hyperhidrosis can be used as a simple screening tool to identify this specific subset of PD patients with dominant autonomic features, although further research is needed.
